# Manipulation of the tumor microenvironment by cytokine gene transfection enhances dendritic cell‐based immunotherapy

**DOI:** 10.1096/fba.2019-00052

**Published:** 2019-11-26

**Authors:** Daluthgamage Patsy Himali Wijesekera, Eiji Yuba, Nadeeka Harshini De Silva, Shun‐ichi Watanabe, Masaya Tsukamoto, Chihiro Ichida, Takeshi Izawa, Kazuyuki Itoh, Ryoji Kanegi, Shingo Hatoya, Jyoji Yamate, Toshio Inaba, Kikuya Sugiura

**Affiliations:** ^1^ Department of Advanced Pathobiology Graduate School of Life and Environmental Sciences Osaka Prefecture University Izumisano Japan; ^2^ Department of Applied Chemistry Graduate School of Engineering Osaka Prefecture University Sakai Japan; ^3^ Department of Integrated Structural Biosciences Graduate School of Life and Environmental Sciences Osaka Prefecture University Izumisano Osaka Japan; ^4^ Research Institute Nozaki Tokushukai Daitou City Japan; ^5^ Present address: Department of Pathobiology Faculty of Veterinary Medicine and Animal Science University of Peradeniya Peradeniya Sri Lanka

**Keywords:** cancer immunotherapy, CD40 ligand, gene therapy, immunity, interferon‐gamma, synthetic vehicle

## Abstract

The tumor microenvironment strongly influences clinical outcomes of immunotherapy. By transfecting genes of relevant cytokines into tumor cells, we sought to manipulate the microenvironment so as to elicit activation of T helper type 1 (Th1) responses and the maturation of dendritic cells (DCs). Using a synthetic vehicle, the efficiency of in vivo transfection of GFP‐cDNA into tumor cells was about 7.5% by intratumoral injection and about 11.5% by intravenous injection. Survival was significantly improved by both intratumoral and intravenous injection of the plasmid containing cDNA of interferon‐gamma, followed by intratumoral injection of DCs presenting the tumor antigens. Also, tumor growth was inhibited by these treatments. A more significant effect on survival and tumor growth inhibition was observed following injection of the plasmid containing cDNA of CD40 ligand, which is a potent inducer of DC‐maturation. Furthermore, the co‐injection of both IFNγ‐ and CD40 ligand‐encoding cDNA‐plasmids, followed by DC treatment, gave rise to further marked and enhancement, including 100% survival and more than 50% complete remission. This treatment regimen elicited significant increases in mature DCs and types of cells contributing to Th1 responses, and significant decreases in immune suppressor cells in the tumor. In the spleen, the treatment significantly increased activities of tumor‐specific killer and natural killer cells, but no alteration was observed in mature DCs or suppressor cells. These results indicate that transfection of these cytokine genes into tumor cells significantly alter the tumor microenvironment and improve the therapeutic results of DC‐based immunotherapy.

**Abbreviations**
Ab: antibody
CTL: cytotoxic T lymphocyte
CD40L: CD40 ligand
DC: dedritic cell
IFNγ: interferon gamma
*i.t*.
: introatumoral, intratumorally
*i.v.*: intravenous, intravenously
MDSC: myeloid derived suppressor cell
NK: natural killer
PD-L: programed cell death ligand
Th1: T helper type 1
Treg: regulatory T cell

## 1 INTRODUCTION

Injection of dendritic cells (DCs) that have been pre‐exposed to tumor antigens has been used to promote T helper type 1 (Th1) responses in patients with different types of tumor. This treatment has almost no toxicity, but the immune responses observed have been transient and overall clinical outcomes have not been particularly successful.^1^ This is primarily due to degradation and dysfunction of DCs by factors derived from the tumor.^2^ On the other hand, the “cancer immunoediting” theory has recently been proposed by Schreiber et al to explain the relation between the immune system and tumor growth, and suggests that the fate of tumors is determined by the immune status in the tumor microenvironment, but not the systemic emvironment.^3^ Moreover patients with tumor microenvironments containing significantly higher numbers of CD8^+^ cytotoxic T lymphocytes (CTLs) and mature DCs, but with fewer regulatory T cells (Tregs), had better prognosis after surgery.^4^, ^5^, ^6^, ^7^, ^8^, ^9^ Based on these observations, we postulated that this manipulation of the microenvironment lead to a favorable clinical outcome. Injection of immune facilitating cytokines into tumor tissue is considered to be an effective method for manipulating the tumor microenvironment. We observed improvements in DC‐based immunotherapy with intratumoral injection of interferon gamma (IFNγ), which is a typical activator of Th1 responses and a promoter of DC maturation.^10^ It was difficult to maintain an effective concentration of the cytokine, however, because of immediate diffusion from the tumors. Repeated administration in order to maintain the concentration unfortunately induced side effects such as inhibition of hematopoiesis, as described previously.^11^

Transfection of the cytokine gene into tumor cells in vivo*,* giving rise to expression from the tumor cells themselves, offers an alternative approach to these limitations: cytokine levels would be maintained in the tumor microenvironment, and systemic diffusion would be prevented because of effective utilization of cytokines by infiltrating immune cells. In the present study, using a synthetic vehicle for in vivo gene transfection, namely pH‐sensitive liposome/lipoplex‐based nonviral vector, we manipulated the microenvironment so as to facilitate DC maturation and activation of Th1 responses. After that, we investigated the therapeutic effects on DC‐based immunotherapy. We thereby demonstrate an effective strategy for improving tumor immunotherapy.

## 2 MATERIALS AND METHODS

### 2.1 Animals and cell line

C3H/He (C3H) female mice (6‐8 weeks old) were purchased from Japan SLC incorporation. The mice were maintained under specific pathogen‐free conditions. After starting experiments mice were monitored daily for weight loss, labored respiration, and any sign of discomfort. There were no any unexpected deaths. The experimental endpoint was 60 days after injecting tumor cells. In the tumor models, mice were immediately euthanized when they exhibited strong distressed signs, such as hunching in the corner of the cage with crumbled hair, rapid weight loss, lack of movement or abnormal respiration, otherwise the longest diameter of the tumor exceeded 2.0 cm. In case of harvesting organs or excising tumor tissue, mice were euthanized immediately before collecting tissues. The mice were humanely euthanized by anesthesia with sodium pentobarbital (200 mg/kg, intraperitoneal injection) followed by cervical dislocation. All of the research staff involved in the animal experiments received the training in animal care and approved by the Animal Experiment Committee of the Osaka Prefecture University. Also, the study protocol was approved by the committee (Approval No. 29‐15 and 30‐6).

The C3H mouse‐derived osteosarcoma line LM8 (RRID:CVCL_6669) was established and maintained by K. Itoh.^12^ The LM8 cells (2 × 10^6^ in 0.1 mL saline) were injected on the back of mice using an insulin‐syringe (29 gauge, 0.5 mL, Nippon Becton Dickinson) under sedated and analgesic condition with medetomidine (50 μg/kg intraperitoneal injection). As described previously,^12^ the LM8 cells characteristically metastasize in the lung from the subcutaneously developed tumor. Thus, the C3H‐LM8 model is more representative of human cancer than the other model. Besides of LM8, YAC‐1, a natural killer (NK)‐sensitive mouse lymphoma line (RIKEN, Cat# RCB‐1165, RRID:CVCL_2244) and BW5147.G.1.4 (BW5147), an AKR/J mouse‐derived lymphoma (ATCC, Cat# TIB‐47, RRID:CVCL_6315) were used in cytotoxicity assay.

### 2.2 Preparation of DCs and tumor antigen

DCs were prepared from bone marrow as described by Akazawa et al^13^ using recombinant mouse granulocyte‐macrophage colony‐stimulating factor (Peprotech). Tumor lysate was used for the preparation of tumor antigens by the freeze‐thaw method.

### 2.3 Preparation of synthetic vehicle for in vivo gene transfection

The synthetic vehicle composed of a cationic lipid enclosing with DNA (lipoplex), which bound with the pH‐sensitive liposome was prepared as described by Sakaguchi et al^14^ and Yuba et al^15^, ^16^ As the cationic lipid, polyamidoamine dendrone lipid (Multifectam^®^, Promega) was used.^17^ The pH‐sensitive liposome was prepared by conjugating liposomes containing egg yolk phosphatidylcholine (Nippon Yuka Kogyo) with 3‐methylglutarylated poly (glycidol) (MGluPG) as the pH sensitive fusogenic polymer.^18^ The MGluPG was bound with transferrin (SIGMA‐Aldrich) to target the tumor cells via its corresponding receptor.^19^ The transfection efficiency of the synthetic vehicle is influenced by the ratio of the [carboxylates of MGluPG] versus [phosphates of DNA] (C/P), and the charge ([number of amines in cationic lipid] relative to [phosphates of DNA]) (N/P). Since the optimal ratio was identified as C/P = 5 and the N/P = 8 in preliminary in vitro experiments, these ratios were used for the in vivo transfection.

### 2.4 Preparation and characterization of cytokine genes

Isolation of RNA, cDNA synthesis, and PCR amplification was carried out as described previously.^20^ In the PCR amplification, primers were designed to amplify a particular nucleotide sequence of mouse IFNγ and CD40 ligand (CD40L) cDNA (NM_008337.4 and NM_011616.2). Sequence recognized by *Xho* I was attached to the forward primer, and sequences recognized by *BamH* I to the reverse primers. The PCR products were then inserted into a PCR‐Blunt vector (Invitrogen) and were amplified in the transformed *E coli* DH5α. After the sequences of inserts were determined, the clone having 100% homology with the desired sequence was selected. The cloned sequence was inserted, utilizing the designed restriction sites, into pcDNA™3.1/myc‐His (‐) (pcDNA) (Invitrogen; Cat# V855‐20) in order to construct the mouse IFNγ or CD40L expression vector.

The pcDNA‐IFNγ cDNA and pcDNA‐CD40L cDNA was transfected in vitro into LM8 cells using the synthetic vehicle as described above. After transfection, cells were selected in a culture with neomycin, G418 (Nacalai Tesque). Expression of IFNγ or CD40L of the selected cells was confirmed in flow cytometry using monoclonal antibodies (mAbs) against mouse IFNγ (clone XMG1.2, Thermo Fisher Scientific; Cat# 12‐7311‐81, RRID:AB_466192) and mouse CD40L (clone MR1, Thermo Fisher Scientific; Cat# 12‐1541‐82, RRID:AB_465887).

### 2.5 Detection of in vivo transfection efficiency

The plasmid containing cDNA encoding the green fluorescent protein (GFP) (pApGFP1‐C1, Clontech Laboratories, Cat# 632 470) was encapsulated into the synthetic vehicle and introduced into tumor‐bearing mice via intratumoral (*i.t.*) or intravenous (*i.v.*) injection through the lateral tail vein due to convenience and minimal stress for mice. Two days after the injection, tumors were excised from euthanized mice for the preparation of paraffin sections. Expression of GFP was detected in immunochemistry using Rabbit‐anti GFP Tag antibody (Ab) (Thermo Fisher Scientific, Cat# A‐6455, RRID:AB_221570) followed by a detection kit containing peroxidase‐labeled goat anti‐rabbit IgG F (ab’)^2^ Ab and 3,3′‐diaminobenzidine tetrahydrochloride (Nichirey Bioscience Inc). Three mice in each injection group were used. Three sections were made from the tumor of each mouse. Cells in three or four high‐power (×400) fields were counted in each section. Total >1000 cells were counted in each tumor. The transfection efficiency was calculated as the number of GFP‐expressing cells divided by total cells counted.

### 2.6 Tumor immunotherapy regimens

Tumor‐bearing mice were treated four times at 7 day intervals. Treatments began 12 days after the injection when the diameter of the tumors was 0.4‐0.6 cm. The synthetic vehicle was prepared to contain 2.5 µg pcDNA inserted with/without cDNA encoding murine IFNγ or CD40L. In some experiments the vehicle enclosed both the IFNγ cDNA‐plasmid and the CD40L cDNA‐plasmid (Comb). The treatment groups were set as follows: [Cytokine DNA (IT) + DC] or [Cytokine DNA (IV) + DC] group, mice were inoculated with the synthetic vehicle enclosing the cytokine cDNA‐plasmid via i.t. or iv route, and were i.t. inoculated with 2 × 10^6^ antigen‐loaded DCs on the following day; [Control DNA (IT) + DC] or [Control DNA (IV) + DC] group, mice were inoculated with the vehicle enclosing empty plasmid and treated with DCs; the [Cytokine (IT)] or [Cytokine (IV)] group, mice were inoculated with the vehicle enclosing cytokine‐gene inserted plasmid but not treated with DCs; [Control (IT)] or [Control (IV)] group, mice were inoculated with the vehicle enclosing empty plasmid; and the [Untreated] group. The treatments were repeated four times at 7 day intervals. The therapeutic effect was evaluated by measuring the tumor size and survival for 60 days. Tumor volume was calculated with the formula: Tumor volume (cm^3^) = (longest diameter) × (short diameter)^2^ × 0.5. Because the volume of tumor was different in each mouse at the start of treatments (day 0), the tumor growth was evaluated as relative tumor volume as described by Herrera‐Abreu et al^21^ The relative tumor volume was calculated by dividing with the tumor volume at day 0. Mice were humanely euthanized by the criteria described above. As LM8 reportedly has high metastatic potential to the lungs,^12^ the dead or euthanized mice were examined for metastases.

### 2.7 Cytotoxicity assay

The activity of the tumor‐specific CTLs and NK cells within the splenic cell populations were evaluated with the ^51^Cr‐released assay as described previously,^22^ using LM8 and YAC‐1 as target cells respectively. BW5147 was used as an MHC‐matched negative control for LM8.

### 2.8 Immunohistochemistry

Cells infiltrating tumor tissues were evaluated by immunohistochemistry using following primary Abs: for IFNγ, Bioss Cat# bs‐0480R‐PE‐Cy7, RRID:AB_11092092; for CD40L, Biorbyt Cat# orb10326, RRID:AB_10746923; for CD8, clone 53‐6.7, Thermo Fisher Scientific Cat# 14‐0081‐81, RRID:AB_467086; for Foxp3, clone FJK‐16s, Thermo Fisher Scientific Cat# 14‐5773‐80, RRID:AB_467575; for Iba1, Wako Pure Chemical Industries Inc, Cat# 019‐19741, RRID:AB_839504; for granzyme B, Spring Bioscience Cat# E2580, RRID:AB_1661202. Staining was performed on the frozen for anti‐CD8 mAb or paraffin sections for the other Abs. Stained products were visualized, as described in Section 2.5, using a detection kit containing peroxidase‐labeled goat IgG F (ab’)^2^ Ab against IgG of species of the primary Abs (Nichirey Bioscience Inc, Cat# 414 351 and 414 341). Experiments were performed using three mice in each treatment group which were euthanized after the treatments followed by immediate tumor excision. Three sections were made from a tumor of each individual mouse. Cells in three or four high‐power (×400) fields were counted in each section. A total of more than 3000 cells were counted in each tumor. Number of positive cells in 1000 cells was evaluated.

### 2.9 Flow cytometry

Population of cells infiltrating in tumor tissue was compared with those in spleen in flow cytometry using a flow cytometer (S3™ Cell Sorter, Bio‐Rad Laboratories Inc). Tumor‐infiltrating cells were isolated as described by Hartley et al^23^ using collagenase type Ⅰ (100 U/mL, Sigma Ardrich). Mature DCs (MHC class II ^high^, CD83^+^), NK cells, myeloid derived suppressor cells (MDSCs; Gr1^+^, CD11b^+^)^24^ and cells expressing the programed cell death ligand 1 (PD‐L1) were detected using following monoclonal Abs: for CD83, clone 3D11, Bio‐Rad Cat# MCA2747A647, RRID:AB_2074745; for MHC class II (MHC II), clone M5/114.15.2 Thermo Fisher Scientific Cat# 12‐5321‐83, RRID:AB_465929; for NK1.1, clone PK136, Thermo Fisher Scientific Cat# 17‐5941‐81, RRID:AB_469478; for Gr‐1, clone RB6‐8C5, Thermo Fisher Scientific Cat# 12‐5931‐83, RRID:AB_466046; for CD11b, clone M1/70, Thermo Fisher Scientific Cat# 22‐7770‐72, RRID:AB_2644066; for PD‐L1, clone MIH6, Abcom Cat# ab80276. Results were analyzed using software, ProSort™ ver. 1.5 (Bio‐Rad).

### 2.10 Statistics

Tumor size differences in the experimental groups were compared using a Tukey Kramer test. Kaplan‐Meier survival curves were compared by the log rank test. A student's *t* test for unpaired samples was used for comparison of two parameters. Incidence of metastasis was compared by *χ* ^2^ test, Differences between groups were determined as significant at *P* < .05.

## 3 RESULTS

### 3.1 Efficiency of gene transfection by the synthetic vehicle

**Figure 1:**
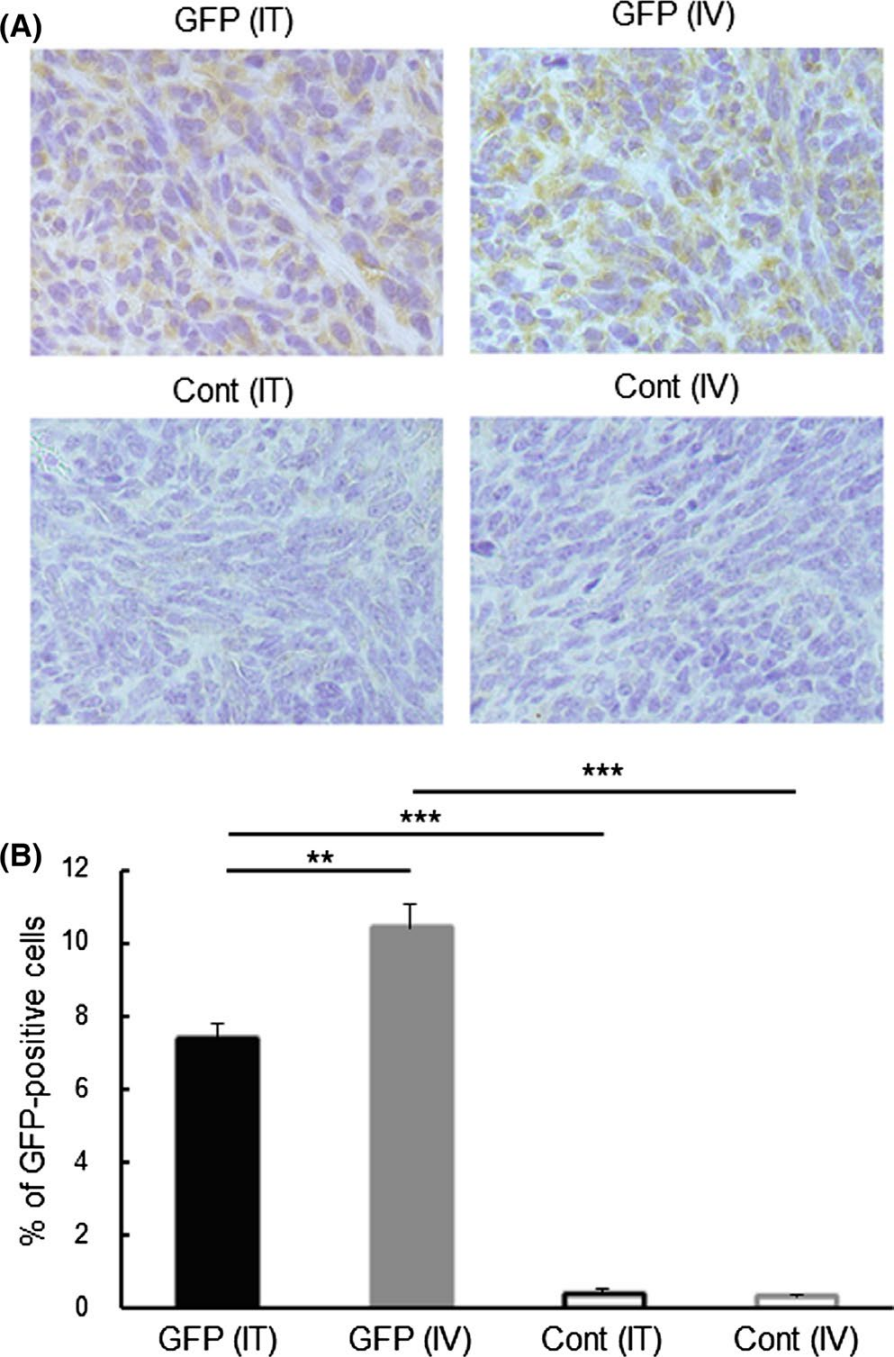
Expression of GFP in tumors growing in mice after intratumoral (IT) or intravenous (IV) injection of the synthetic vehicle containing a GFP cDNA‐plasmid. Tumor tissues were collected from the mice 2 d after the indicated treatments. Expression of GFP in the tumor cells was examined by immunohistochemistry. (A) Typical photos of tumors collected from the indicated treatment groups. Expression of GFP is shown as brown color products. To count the GFP‐expressing cells, nuclei were stained with Mayer's hematoxirine. (B) The percentage of GFP‐expressing cells in tumors of the indicated treatment. Three mice in each injection group were used. Three sections were made from the tumor in each mouse. Cells in three or four high‐power (×400) fields were counted in each section. A total >1000 cells were counted in each tumor. Results are expressed as mean ± SE of three tumors. ***P < .001, **P < .01

The efficiency of gene transfection by the synthetic vehicle was determined using a plasmid encoding GFP cDNA. The efficiency of GFP expression was 7.6 ± 0.4% by *i.t.* injection of the vehicle including GFP plasmid and 11.5 ± 0.7% by *i.v.* injection. Although the efficiency of expression increased significantly compared with that after injection of the vehicle including control plasmid by either route, *i.v.* injection was more efficient than *i.t.* injection (Figure 1). The strength of the expression was almost same in whole area by *i.v.* injection, but different from area to area by *i.t.* injection (Figure S1). Liver and bone marrow cells were also examined for expression of GFP. No expression was found in these organs and cells (data not shown).

### 3.2 Effect of in vivo transfection of IFNγ gene on DC‐based immunotherapy

**Figure 2:**
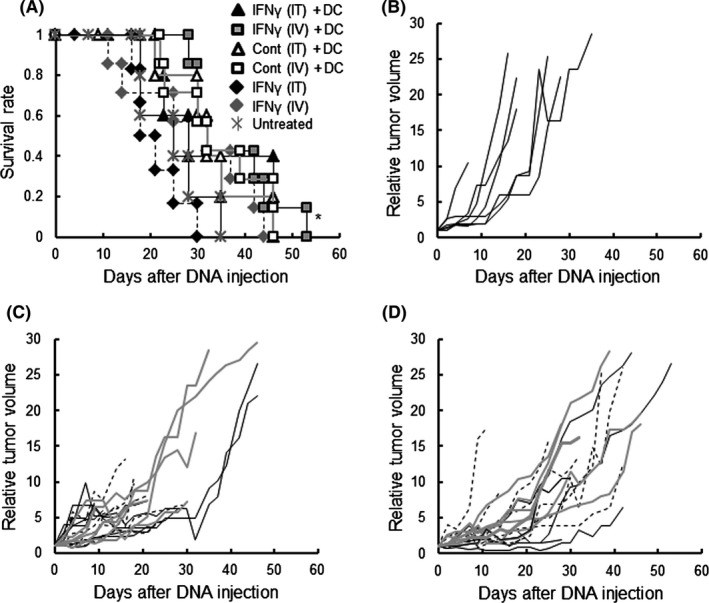
Effect of IFNγ gene‐transfection on DC‐based therapy. (A) Survival of mice with the indicated treatments. (B) The relative tumor volume (with the volume on day 0 set at 1) of each mouse in the [Untreated] group. (C) The relative tumor volume of each mouse in the intratumoral (IT) gene‐administered group. Black solid lines indicate relative tumor volumes of the [IFNγ (IT) +DC] group. Black dotted lines indicate relative tumor volumes of the [IFNγ (IT)] group. Gray solid lines indicate the relative tumor volumes of the [Cont (IT) + DC] group. (D) The relative tumor volume of each mouse in the intravenous (IV) gene‐administrated group. Black solid lines indicate relative tumor volumes of the [IFNγ (IV) +DC] group. Black dotted lines indicate relative tumor volumes of the [IFNγ (IV)] group. Gray solid lines indicate relative tumor volumes of the [Cont (IV) + DC] group. Experiments were performed independently three times using a total of seven mice in each group. All mice were humanely euthanized according to the criteria in Section 2. *P < .01, vs the [Untreated]

It was confirmed IFNγ was expressed in tumor cells growing in mice after injection of the vehicle containing IFNγ gene (Figure S2). As shown in Figure 2A, no mice survived to the experimental end point. The [IFNγ (IV) + DC] group exhibited a significantly longer survival compared to the [Untreated] group, but the [IFNγ (IT) + DC] group did not. As shown in Figure 2B‐D, the growth of LM8 tumor in the [IFNγ + DC] groups was less than in the [Untreated] group, but was not very different from that in the [IFNγ] and [[Cont + DC] groups. Metastases in the lung were found as early as 4 days after the beginning of treatment (that is, 16 days after the tumor injection) in the [IFNγ (IV)] treated mouse. The incidence of metastasis in the [IFNγ (IV) + DC] group did not differ from that in the other groups (Table 1).

**Table 1:** Lung metastasis in the LM8‐inoculated mice

Treatment group	Mice with metastasis	Total mice examined	Incidence (%)
IFNγ (IT) +DC	4	7	57
IFNγ (IV) +DC	5	7	71
IFNγ (IT)	7	7	100
IFNγ (IV)	6	7	86
Cont (IT) +DC	5	7	71
Cont (IV) +DC	5	7	71
Untreated	6	7	86
CD40L (IT) +DC	5	8	63
CD40L (IV) +DC	4	8	50
CD40L (IT)	6	8	75
CD40L (IV)	6	8	75
Cont (IT) +DC	5	8	63
Cont (IV) +DC	5	8	63
Untreated	7	8	88
Comb (IT) +DC	0	6	0†
Comb (IV) +DC	0	6	0‡
Comb (IT)	3	6	50
Comb (IV)	2	6	33
Cont (IT) +DC	4	6	67
Cont (IV) +DC	4	6	67
Untreated	6	6	100

^†^ *P* < .01 vs “Utreated” group and *P* < .05 vs “Cont (IT) +DC” group by *χ* ^2^ test.

^‡^ *P* < .01 vs “Utreated” group and *P* < .05 vs “Cont (IV) +DC” group by *χ* ^2^ test.

### 3.3 Effect of in vivo transfection of CD40L gene on DC‐based immunotherapy

**Figure 3:**
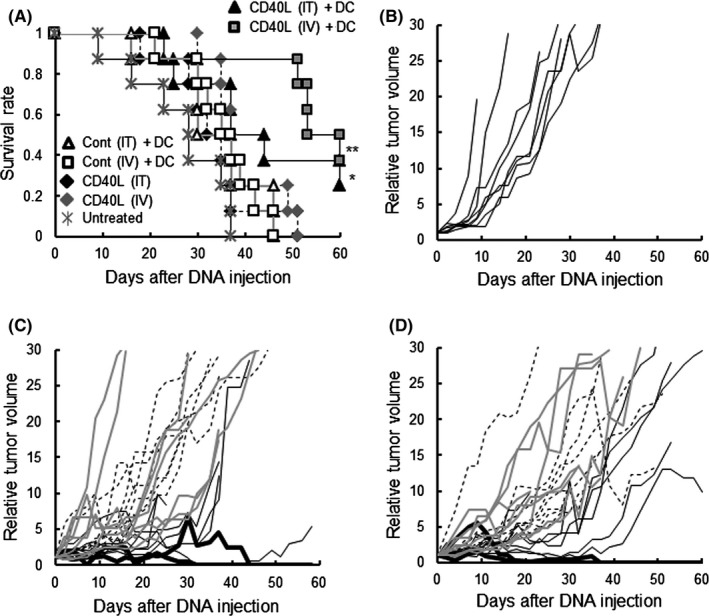
Effect of CD40L gene‐transfection on DC‐based therapy. (A) Survival of mice with the indicated treatments. (B) The relative tumor volume (with the volume on day 0 set at 1) of each mouse in the [Untreated] group. (C) The relative tumor volume of each mouse of the intratumoral (IT) gene‐administered group. Black solid lines indicate relative tumor volumes of the [CD40L (IT) +DC] group. Black dotted lines indicate relative tumor volumes of the [CD40L (IT)] group. Gray solid lines indicate relative tumor volumes of the [Cont (IT) + DC] group. (D) The relative tumor volume of each mouse in the intravenous (IV) gene‐administered group. Black solid lines indicate those of the [CD40L (IV) +DC] group. Black dotted lines indicate those of the [CD40L (IV)] group. Gray solid lines indicate those of the [Cont (IV) + DC] group. Experiments were performed independently three times, using a total of eight mice in each group. All mice were humanely euthanized according to the criteria in Section 2. *P < .05, vs the [Untreated] group. **P < .01, vs the [Untreated] and [Cont (IV) + DC] groups

It was confirmed that CD40L was expressed in tumors growing in mice after injection of the vehicle containing CD40L gene (Figure S3). As it has been reported that CD40L induces maturation of DC for enhancing immune responses against cancer,^25^ the CD40L gene was used instead of IFNγ, and the therapeutic effect was examined. The in vitro preliminary experiments confirmed that the CD40L expressed by the gene transfection elicited DC maturation, which was demonstrated with morphological change and expression of surface molecules (data not shown). As shown in Figure 3A, two of eight mice in the [CD40L (IT) + DC] group and three of eight mice in the [CD40L (IV) + DC] group survived to the end point. The [CD40L (IT) + DC] group survived significantly longer than the [Untreated] group. Moreover the [CD40L (IV) + DC] group survived significantly longer than the [Untreated] group and also the [Cont (IV) +DC] group. In addition, as shown in Figure 3B‐D, growth of the LM8 tumor in the [CD40L + DC] groups was less than in the [Untreated], [CD40L] and [Cont + DC] groups. One of the eight mice (12.5%) in the [CD40L (IT) + DC] group, and two mice (25%) in the [CD40L (IV) + DC] group, were in complete remission at the end point. However, the incidence of metastasis of the both [CD40L + DC] groups was not different from that in the other groups (Table 1).

### 3.4 Effect of the in vivo transfection of IFNγ and CD40L genes on DC‐based immunotherapy

**Figure 4:**
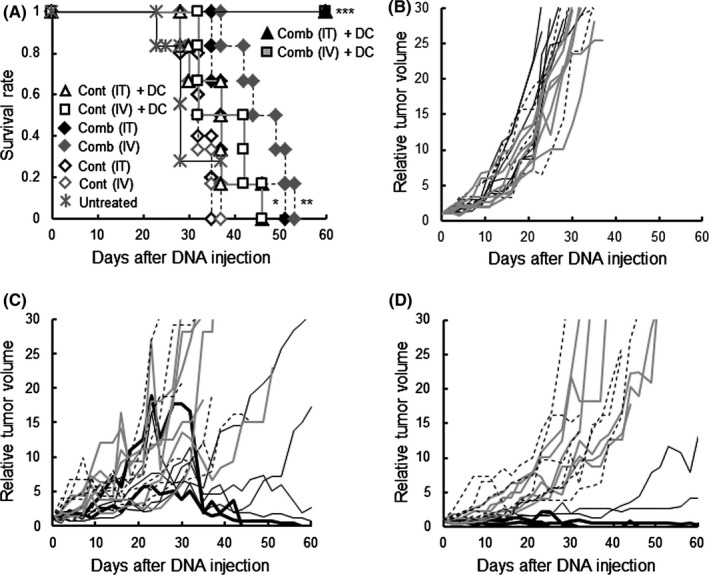
Effect of IFNγ and CD40L (Comb) gene‐transfection on DC‐based therapy. (A) Survival of mice with the indicated treatments. (B) The relative tumor volume (with the volume on day 0 set at 1) of each mouse in the control groups. Black solid lines indicate the relative tumor volumes of the [Untreated] group; black dotted lines indicate those of the [Cont (IT)] group; gray solid lines indicate those of the [Cont (IV)] group. (C) The relative tumor volume of each mouse in the intratumoral (IT) gene‐administrated group. Black solid lines indicate relative tumor volumes of the [Comb (IT) +DC] group. Black dotted lines indicate those of the [Comb (IT)] group. Gray solid lines indicate those of the [Cont (IT) + DC] group. (D) The relative tumor volume of each mouse in the intravenous (IV) gene‐administrated group. Black solid lines indicate relative tumor volumes of the [Comb (IV) +DC] group. Black dotted lines indicate those of the [Comb (IV)] group. Gray solid lines indicate those of the [Cont (IV) + DC] group. Experiments were performed independently three times using a total of six mice in each group. All mice were humanely euthanized according to the criteria in Section 2. *P < .05, vs the [Cont (IT)] group. **P < .05 vs the [Untreated] group, and *P* < .01, vs the [Cont (IV)] group. ***P < .05 vs the [Untreated] group, and *P* < .005, vs the [Cont (IT) +DC] group or the [Cont (IV) +DC] group, respectively

To investigate synergistic effects of combining IFNγ and CD40L, the vehicle containing plasmids for both cytokine cDNAs (Comb) was administered to tumor‐bearing mice. As shown in Figure 4A, the [Comb (IT) + DC] and [Comb (IV) + DC] groups had 100% survival. Survival in the [Comb + DC] group was significantly longer than in the [Untreated] group and also in the corresponding [Cont + DC] group. In the groups with gene transfection but without DC treatment, survival in the [Comb (IV)] group was significantly longer than in the [Untreated] and [Cont (IV)] groups, and survival in the [Comb (IT)] group was significantly longer than in the [Cont (IT)] group. As shown in Figure 4B, tumor growth in the [Untreated], [Cont (IT)] and [Cont (IV)] groups was not different or faster than in the other groups. As shown in Figure 4C, tumor size in the [Comb (IT) + DC] group, as in the [Comb (IT)] and [Cont (IT) + DC] groups, increased by day 32, then decreased by days 35 to 40. Tumors in three of the six mice (50%) in the [Comb (IT) + DC] group continued to reduce in size by the end point, and mice were in complete remission. As shown in Figure 4D, the tumor size in the [Comb (IV) + DC] group, which was different from that in the [Comb (IV)] and [Comb (IV) + DC] groups, did not significantly increase for over 40 days; thereafter, the tumor in four of the six mice (67%) continued to decrease, and had disappeared completely by the end point. As shown in Table 1, lung metastasis was not detected in the [Comb (IT) + DC] or [Comb (IV) + DC] groups. The incidence of metastasis in both groups was significantly lower than in the [Cont + DC] groups and the [Untreated] group.

### 3.5 Effect of the IFNγ and CD40L gene‐transfection with DC‐therapy on immune responses in the tumor microenvironment

**Figure 5:**
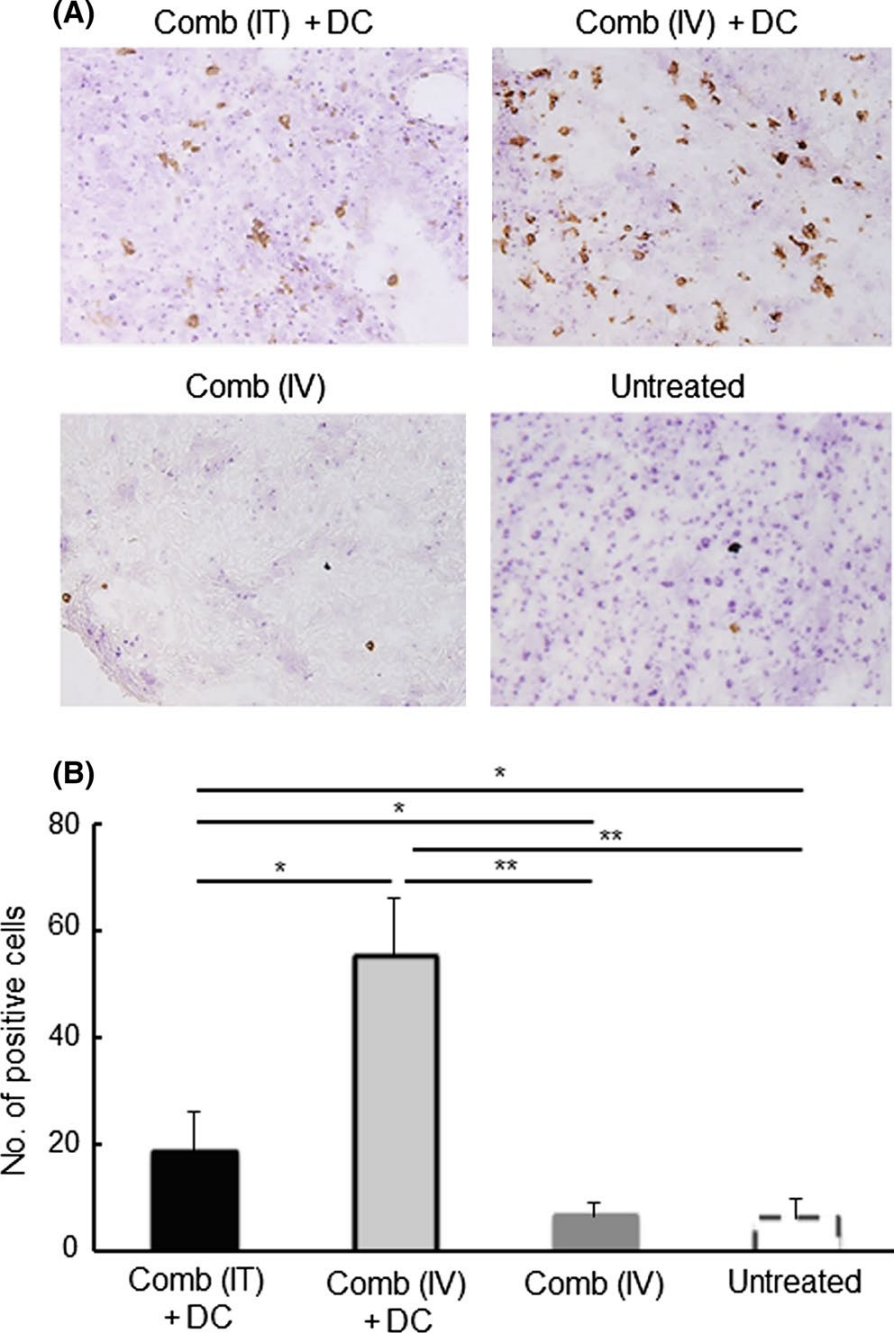
Effect of IFNγ and CD40L (Comb) gene‐transfection therapy on the infiltration of CD8^+^ cells into LM8 tumors. Tumor tissue was collected from mice with the indicated treatments at 7 d after the second treatment. Immunohistochemistry was performed to detect CD8^+^ cells present in the tumor tissue. (A) Typical photos of tumors collected from the indicated treatment groups are shown. Expression of CD8 is shown as brown color products. (B) Counts of CD8^+^ cells in 1000 cells in tumors are shown. Results are expressed as mean ± SE. The experiment was performed using three mice in each treatment group. All mice were humanely euthanized according to the criteria in Section 2. *P < .05, **P < .01 by the Tukey‐Kramer test

To investigate the relation between therapeutic results and immune status in the tumor microenvironment, as in the report by Gao et al,^6^ CD8^+^ cells and FoxP3^+^ cells that had infiltrated were examined in the tumor tissue of the treated groups, all of which exhibited significantly longer survival than the [Untreated] group. As shown in Figure 5, a significantly higher concentration of CD8^+^ cells was present in tumors of the [Comb (IV) + DC] group than in the other groups. The [Comb (IT) + DC] group had a significantly higher concentration of CD8^+^ cells than the [Comb (IV)] and [Untreated] groups. As shown in Figure 8, in contrast, significantly lower proportion of FoxP3^+^ Tregs was present in tumors of the [Comb (IV) + DC] group than in the [Comb (IV)] and [Untreated] groups. The tumors of the [Comb (IT) + DC] group had significantly lower concentrations of Tregs than in the [Untreated] group. To further clarify the effect of expression of the cytokines and the inoculation of exogenous DCs on the immune status of the tumor microenvironment, immune cells that infiltrated into the tumor were compared in the four *i.v.* treated groups. As shown in Figures 7 and 8 (Figures S4, S5, S7, and S8), tumors in the [Comb (IV) + DC] group had significantly higher concentrations of mature DCs (MHC II ^high^, CD83^+^ cells), NK cells and Iba 1^+^ macrophages than in the other three groups, and a significantly higher concentration of granzyme B^+^ killer cells than in the [Cont (IV) + DC] and [Cont (IV)] groups (Figure 8). In contrast, tumors of the [Comb (IV) + DC] group had significantly lower concentrations of MDSCs and cells expressing PD‐L1 than in the other groups (Figure 7; Figures S6 and S7). Compared with tumors of the [Cont (IV) + DC] group, those of the [Comb (IV)] group, which displayed longer survival, had significantly lower concentrations of MDSCs and PD‐L1‐expressing cells. Since expression of PD‐L1 was not detected on LM8 cells in tumors by immunohistochemistry, the PD‐L1‐expressing cells are believed to be monocytes or macrophages infiltrating into the tumor.^23^

### 3.6 Effect of the IFNγ and CD40L gene‐transfection therapy on systemic immune responses

To investigate the systemic anti‐tumor immune responses elicited by the combined cytokine gene treatment, spleen cells were collected 7 days after the second treatment (on day 14), and were examined in cytotoxic assays. As shown in Figure 9A, spleen cells from the [Comb (IV) + DC] group exhibited significantly greater cytotoxic activity against LM8 tumor cells than those from the other groups. However, spleen cells from the [Comb (IT) + DC] and [Comb (IV)] group did not have significantly higher activity than those from the [Untreated] group. As shown in Figure 9B, spleen cells from any treated group exerted negligible cytotoxic activity against the MHC‐matched and a different type of tumor line, BW5147. As shown in Figure 9C, the treated groups all had significantly higher cytotoxic activity against YAC‐1 (NK activity) than those from the [Untreated] group. Spleen cells from the [Comb (IV) + DC] group did not exhibit significantly stronger NK activity than those from the [Comb (IT) + DC] and [Comb (IV)] groups. As shown in Figure 10, spleens in the [Comb (IV) + DC] and [Comb (IV)] groups had a higher proportion of NK cells than in the [Cont (IV)] group. In contrast to results in tumors, the proportions of mature DCs, MDSCs and PD‐L1expressing cells were not different significantly in the four *i.v.* treated groups.

## 4 DISCUSSION

The immune status of the tumor microenvironment has a major influence on the efficacy of cancer immunotherapy. In this study, we facilitated DC maturation and subsequent activation of the Th1 response within the tumor microenvironment by transfection of two cytokine genes, using a synthetic vehicle. We then examined downstream effects on DC‐based immunotherapy.

The synthetic vehicle comprised of a cationic lipid combined with pH‐sensitive liposomes exerted approximately 10% of tumor cells expressed the encapsulated cDNA by both *i.t.* and *i.v.* inoculation (Figure 1). Moreover, no transfection product was found in normal cells in the organs in which drugs are often trapped, or in which active cell‐proliferation occurs. The synthetic vehicle has 100‐200 nm‐size^14^ and, as a nanoparticle, is able to accumulate in tumor tissue by “the enhanced permeability and retention effect”.^26^ In addition, the tumor specificity of the synthetic vehicle is enhanced by the transferrin epitopes which target the corresponding receptor that is generally expressed on tumor cells.^14^ Furthermore, the pH‐sensitive polymer, MGluPG, which induces fusion with endosomal membranes due to the low pH of the endosome, effectively releases the encapsulated DNA into cytoplasm prior to lyososmal degradation.^14^, ^15^, ^16^, ^27^ These mechanisms are together believed to elicit tumor‐specific and more effective gene transduction by *i.v.* inoculation of the vehicle. The *i.v.* inoculation of the vehicle with cytokine genes actually elicited stronger therapeutic effects (Figures 2, 3, 4). The results extend, moreover, to possible treatment of visceral tumors or metastasis. Using an adenovirus vector, the CD40L gene has been effectively transfected into tumor cells and showed therapeutic results.^28^, ^29^, ^30^ Because of immunogenicity,^31^ however, gene transduction by the viral vector is effective only by *i.t.* injection, and not by *i.v.* injection. Moreover there were sometimes significant problems with the viral vectors relating to pathogenicity.^32^ No toxicity was found by histopathological examination in the present study. Based on these observations, it is clear that the synthetic vehicle is a promising candidate for gene transduction in the clinical treatment.

In the context of survival, the [CD40L (IV) +DC] group and the [Comb + DC] groups survived significantly longer than the [Cont (IV) +DC] group or the [Cont + DC] groups (see Figures 3A and 4A). Moreover, the [Comb] groups survived significantly longer than the [Control] groups (Figure 4A). CD40L is a typical cytokine which induces the maturation of DCs.^25^ Indeed, tumors in the [Comb (IV) +DC] group had a significantly larger number of CD83^+^ mature DCs than tumors in the [Cont (IV) +DC] group (Figure 7). Based on these observations, we strongly suggest that modification of the microenvironment so as to promote DC maturation is critical to the improvement of therapeutic outcomes.

In the IFNγ cDNA‐treated groups, the [IFNγ (IV) +DC] group alone significantly exerted survival relative to the untreated group. However, no mice receiving the treatment survived to the desired end point (Figure 2). Although IFNγ is a potent activator of the Th1 system, it also induces Tregs^33^ and myeloid derived suppressor cells (MDSC).^34^ These undesirable effects may be responsible for the limited success of the IFNγ therapy group. Compared with the IFNγ gene treatment, transfection of the CD40L gene gave greater enhancement of DC therapy, such that 25% of the *i.t.* treated recipients and 38% of *i.v.* treated recipients survived to the end point (Figure 3), although the efficiency of the gene expression of CD40L was similar to that of the IFNγ (Figures S2 and S3). CD40L efficiently activates cellular immunity via activation of DCs.^35^ Furthermore, since CD40L is normally membrane‐bound, the effects of CD40L are believed to be longer‐lasting than those of IFNγ. Moreover, induction of Treg and MDSC has not been reported with CD40L. CD40L may therefore elicit a more potent therapeutic effect than IFNγ. Using both IFNγ and CD40L genes, a further pronounced effect was obtained, exceeding that using CD40L gene alone (Figure 4). In in vitro experiments, it was reported that IFNγ and CD40L exerted synergic effects^36^ in which IFNγ directly activates Th1 responses, whereas CD40L functions indirectly by enhancing DC function. The difference in the point of action may explain the synergistic effect. We believe this is the first report to demonstrate the synergic effects in in vivo gene transduction. The *i.t.* injection group displayed increases in tumor size during the four treatments, but four of the six tumors reduced thereafter, whereas in the *i.v.* injection group the tumors significantly decrease during treatment (Figure 4). We found that the strength of gene expression was different in area with *i.t.* injection (Figure S1). Expression was therefore believed to be strong around the injection site, inducing immune responses so as to destroy tumor cells, but not in the other areas where tumor cells continued to proliferate initially, causing the tumor size to increase. As time passed, however, the immune responses might have come to extend to the entire tumor and reduced it.

**Figure 6:**
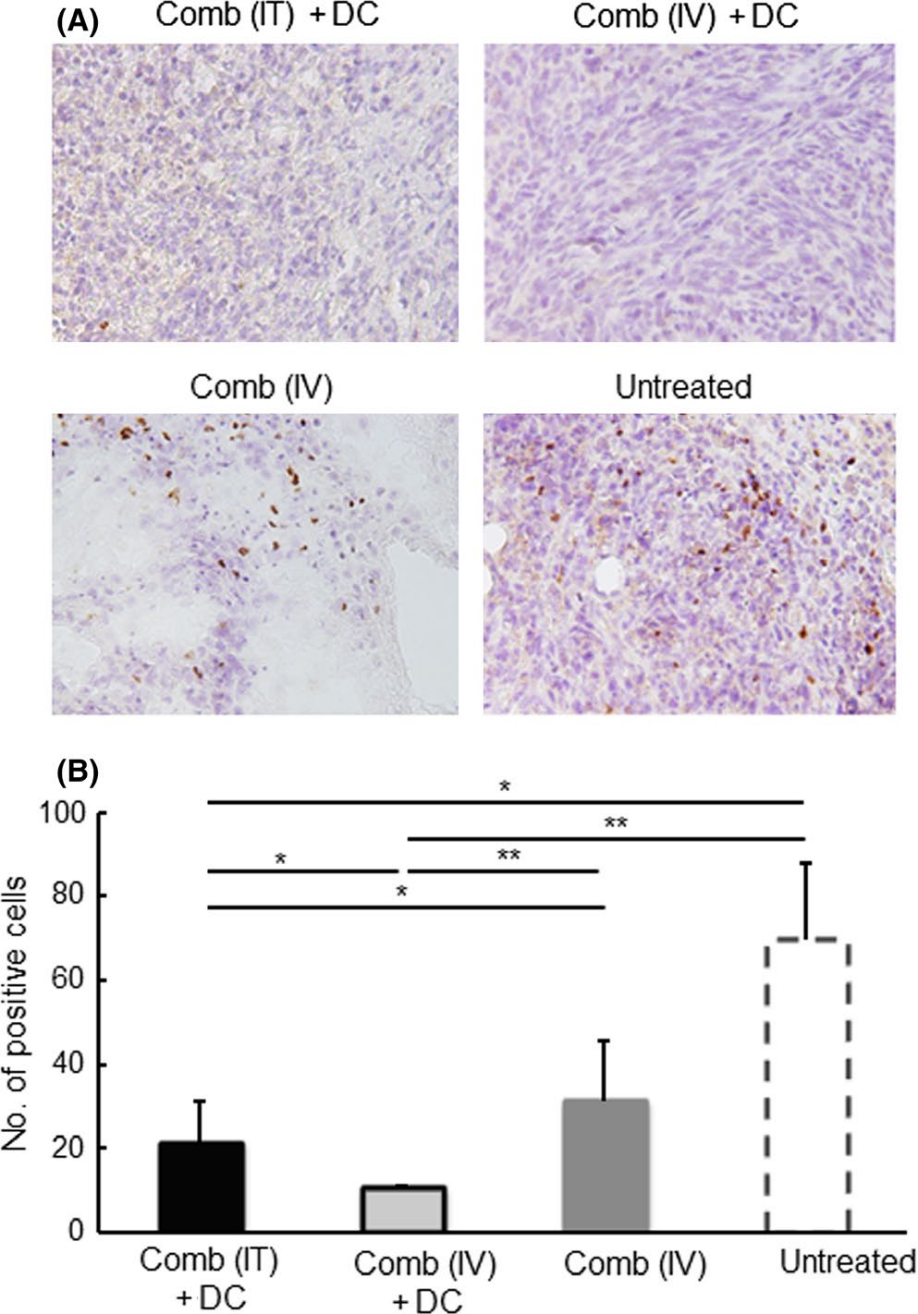
Effect of IFNγ and CD40L (Comb) gene‐transfection therapy on the infiltration of FoxP3^+^ cells into the tumors. Tumor tissue was collected from mice with the indicated treatments at 7 d after the second treatment. Immunohistochemistry was performed to detect FoxP3^+^ cells present in the tumor tissue. (A) Representative photo data of the tumors collected from the indicated treatment groups are shown. Expression of FoxP3 is shown as brown color products. (B) Counts of FoxP3^+^ cells in 1000 cells in tumors are shown. Results are expressed as mean ± SE. The experiment was performed using three mice in each treatment group. All mice were humanely euthanized according to the criteria in Section 2. *P < .05, **P < .01 by the Tukey‐Kramer test

**Figure 7:**
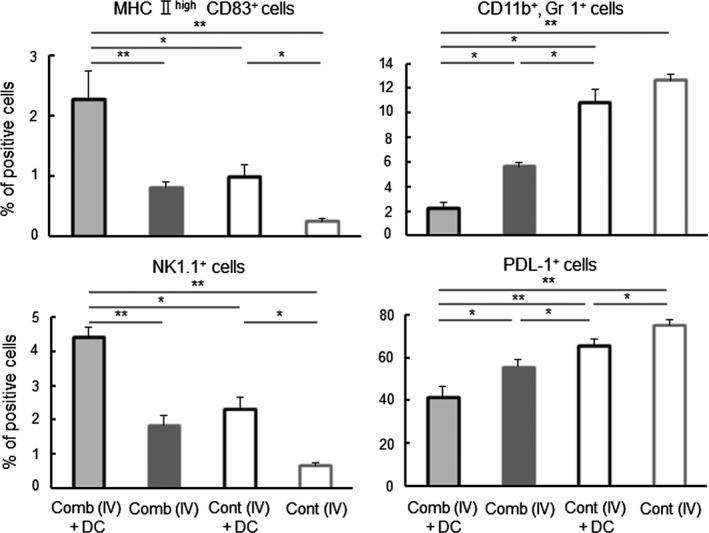
Effect of IFNγ and CD40L (Comb) gene‐transfection therapy on tumor‐infiltrating cells. Tumor tissue was collected from mice with the indicated treatments at 7 d after the second treatment. Mature DCs (MHC II ^high^ CD83^+^ cells), MDSCs (CD11b^+^, Gr1^+^ cells), NK (NK1.1^+^) cells and PD‐L1^+^ cells in the tumor tissues were detected in FCM. Results are expressed as mean ± SE. The experiment was performed using three mice in each treatment group. All mice were humanely euthanized according to the criteria in Section 2. *P < .05, ** *P* < .01 by the Tukey‐Kramer test

**Figure 8:**
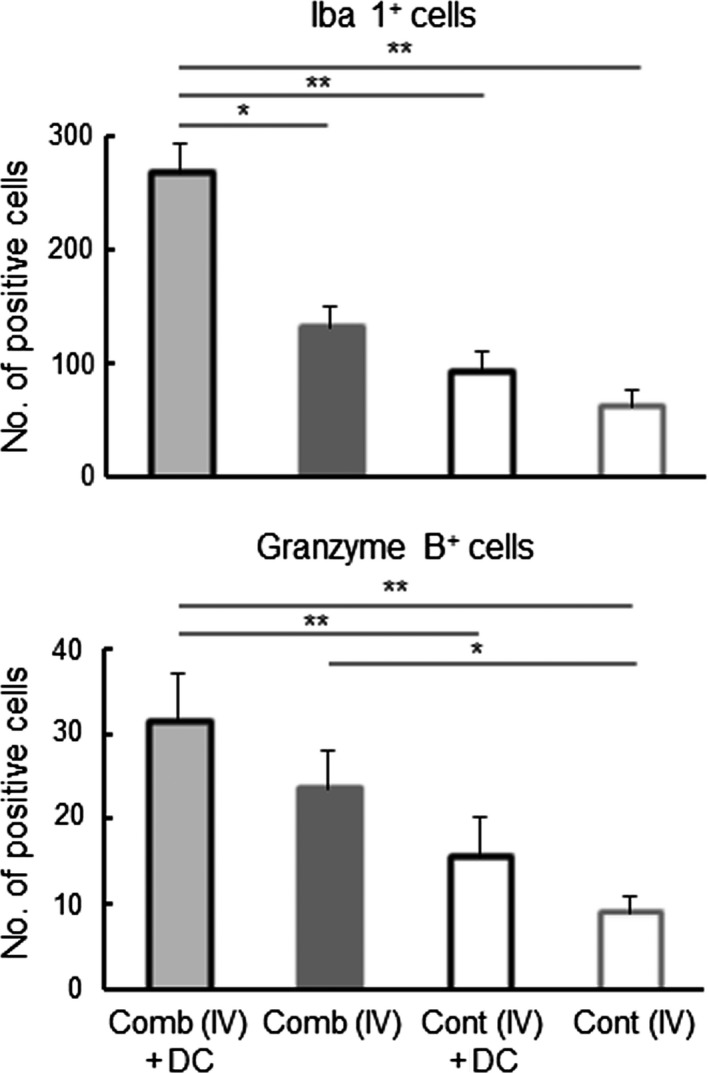
Effect of the IFNγ and CD40L (Comb) gene‐transfection therapy on tumor‐infiltrating cells. Tumor tissue was collected from mice with the indicated treatments at 7 d after the second treatment. The Iba 1^+^ activated macrophage and granzyme B^+^ killer cells in the tumor tissue were detected by immunohistochemistry. Results are expressed as mean ± SE. The experiment was performed using three mice in each treatment group. All mice were humanely euthanized according to the criteria in Section 2. *P < .05, **P < .01 by the Tukey‐Kramer test

**Figure 9:**
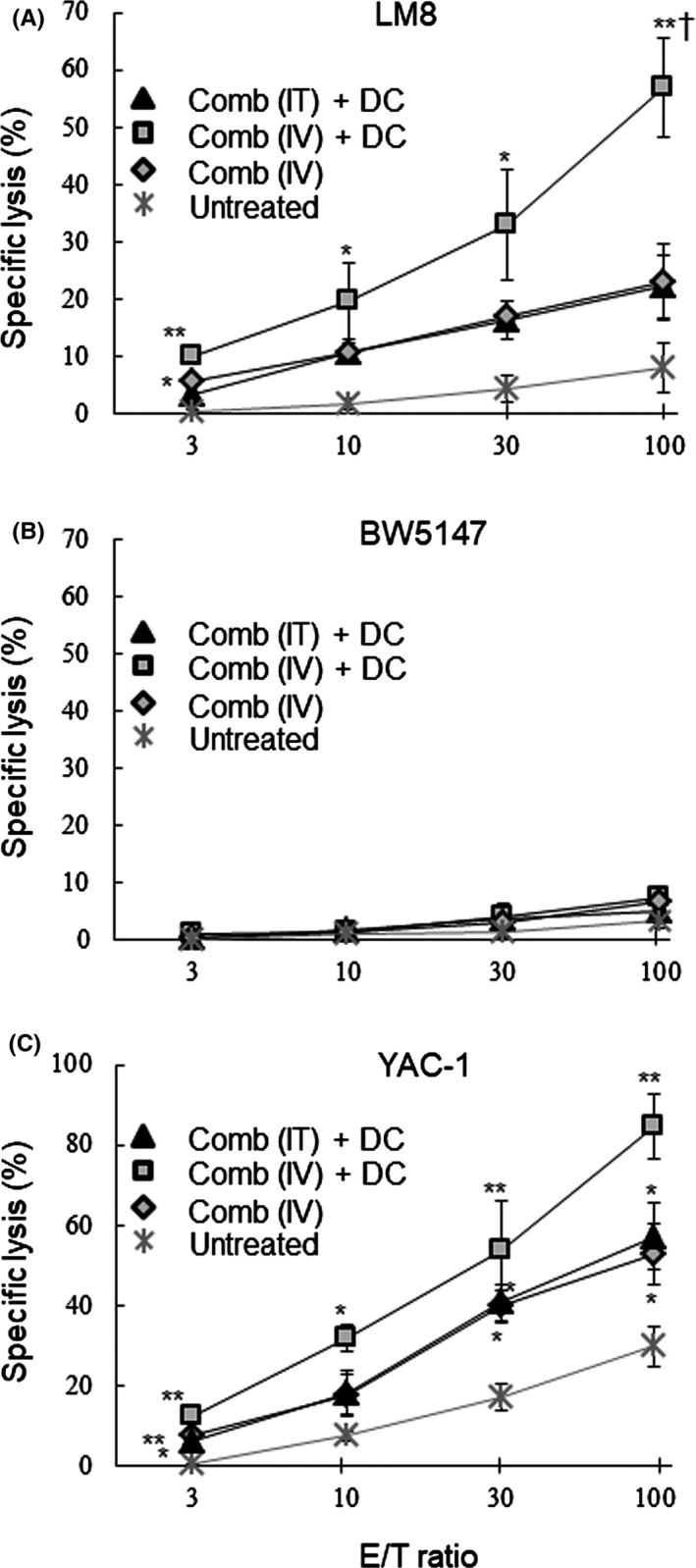
Effect of IFNγ and CD40L (Comb) gene‐transfection therapy on systemic immune responses. Spleen cells were collected from the LM8 tumor‐bearing mice with the indicated treatments at 7 d after the second treatment. Cytotoxic activity of the cells against LM8 (A), BW5147 (B), and YAC‐1 (C) was evaluated in vitro. The experiments were repeated twice. Each experiment was performed using three mice in each treatment group. A typical result is shown. All mice were humanely euthanized according to the criteria in Section 2. Results are expressed as mean ± SE. *P < .05, **P < .01 vs the [Untreated] group, ^†^ *P* < .05 vs the [Comb (IT) + DC] and [Comb (IV)] group at the E/T ratio

**Figure 10:**
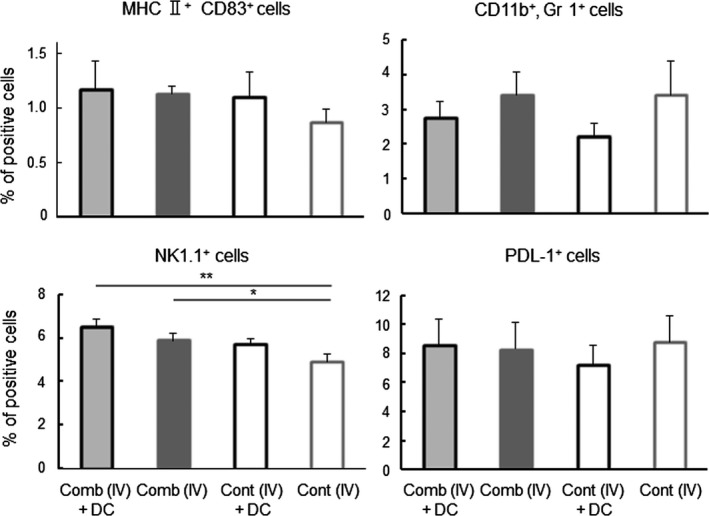
Effect of IFNγ and CD40L (Comb) gene‐transfection therapy on spleen cells. Spleen was collected from mice with the indicated treatments at 7 days after the second treatment. Mature DCs (MHC II ^high^ CD83^+^ cells), MDSCs (CD11b^+^, Gr1^+^ cells), NK (NK1.1^+^) cells and PDL‐1^+^ cells in the tumor tissues were detected in FCM. Results are expressed as mean ± SE. The experiment was performed using three mice in each treatment group. All mice were humanely euthanized according to the criteria in Section 2. *P < .05, **P < .01 by the Tukey‐Kramer test

A significantly greater number of CD8^+^ CTLs, and significantly fewer FoxP3^+^ Tregs, were found in tumors of the groups receiving the cytokine gene transduction and DC treatment, which yielded significantly better results in tumor therapy (Figures 5 and 6). These findings suggest that immune responses against the tumor were significantly enhanced in the tumor microenvironment of these groups. Moreover, the group experiencing gene transduction plus DC had significantly higher concentrations of mature DCs, killer cells and activated macrophages, but had significantly lower concentrations of MDSCs and PD‐L1 expressing cells in the tumor (Figures 7 and 8). Although activity and concentration of NK cells was significantly increased in the spleen by the gene transduction plus DC treatment, concentrations of the other cell populations were not changed (Figures 9 and 10). Taken together with the results of previous studies,^4^, ^6^, ^7^ these findings strongly indicate that the immune status in the microenvironment, although not in the wider system, significantly influence to the results of tumor therapy.

DCs maturating and activated in the tumor microenvironment may ultimately move to the lymph nodes or the spleen, and activate tumor‐specific CTLs and NK cells systemically. Indeed, CTL and NK activity was significantly enhanced systemically in the [Comb + DC] groups and in the [Comb (IV)] group (Figure 9), in which survival was significantly longer than in groups with unmodified microenvironment (Figure 4A). The [Comb + DC] groups had a significantly lower incidence of metastasis (Table 1). Although Joyama et al^37^ found that the nodule number and size of the lung metastases was not related to the size of subcutaneous tumors, the incidence of metastasis was a critical factor for survival in this study (see Figures 2A, 3A, 4A and Table 1). Based on these findings, we suggest that enhancement of tumor immunity in the microenvironment is related to the promotion of systemic immunity, which acts to prevent metastasis and contributes to the longer survival times.

Taken together, the results presented here indicate that transfection of the gene of the corresponding cytokines, using the synthetic vehicle described here, effectively give rise to a microenvironment which not only promotes the maturation and activation of DCs inoculated and enhances Th1 responses, but also reduces the generation of suppressor cells. The microenvironment also significantly enhanced systemic immune responses against the tumor, and ultimately elicited significant improvement in DC‐based immunotherapy.

## CONFLICT OF INTEREST

The authors have no conflict of interest.

## AUTHOR CONTRIBUTIONS

K. Sugiura designed research; E. Yuba contributed new reagents; K. Itoh contributed research tool; DPH Wijesekera, NH De Silva, T. Izawa, S. Hatoya and K. Sugiura analyzed data; DPH Wijesekera, NH De Silva, E. Watanabe, M. Tsukamoto, C. Ichida, and R. Kanegi performed research; DPH Wijesekera, NH De Silva and K. Sugiura wrote the paper; E. Yuba, K. Itoh, J. Yamate T. Inaba supervised the research and edited the paper.
