# Supplementary figures and images for: Manipulation of the tumor microenvironment by cytokine gene transfection enhances dendritic cell‐based immunotherapy
Source: FASEB Bioadv. 2019 Nov 26;2(1):5–17. doi: 10.1096/fba.2019-00052 (PMC6996313; doi:10.1096/fba.2019-00052)

**Expression of GFP**

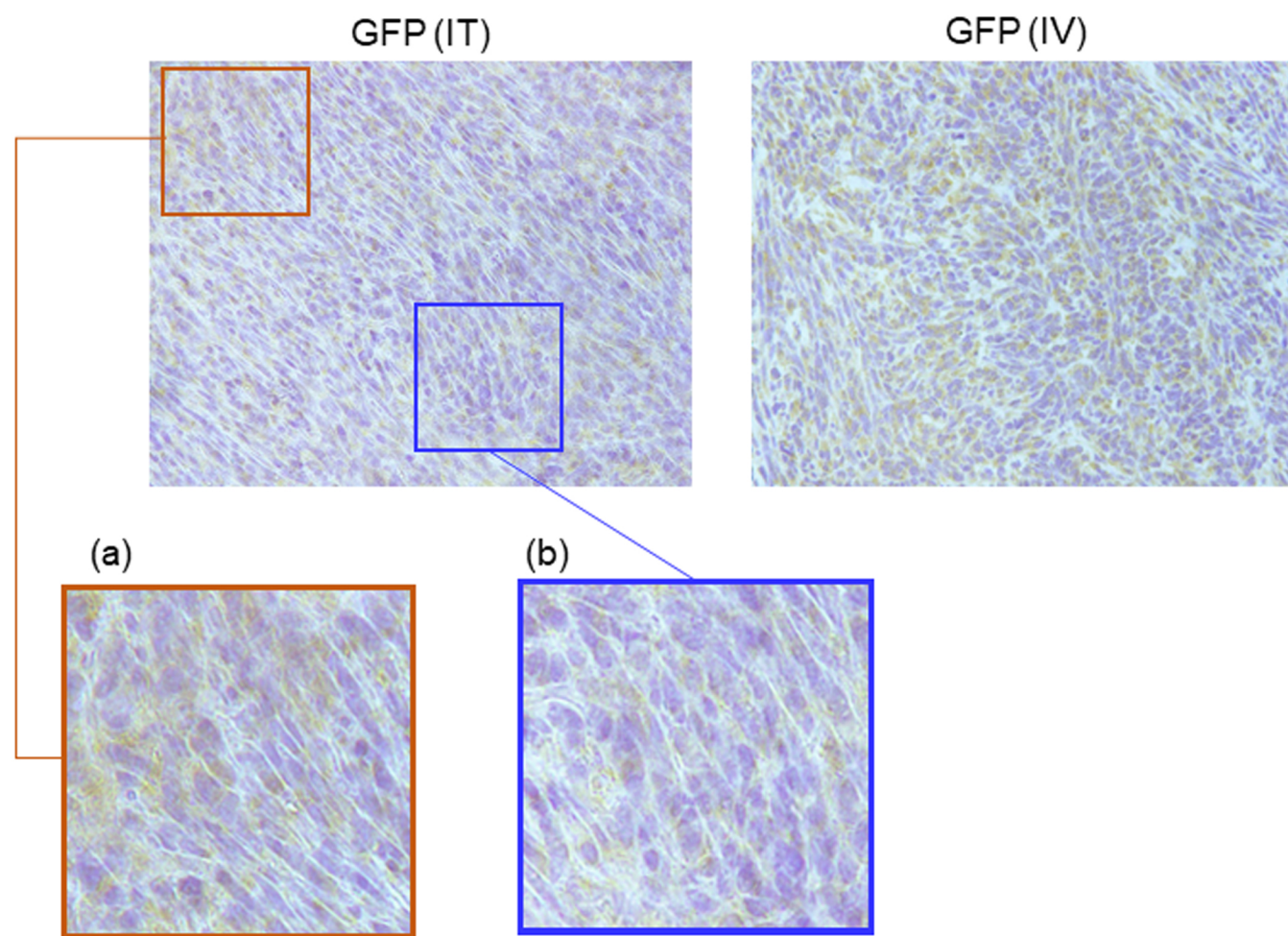

Supplement: Supplementary file 1 [file FBA2-2-5-s001.pdf]

**Expression of IFN $\gamma$**

IFN $\gamma$  (IV)

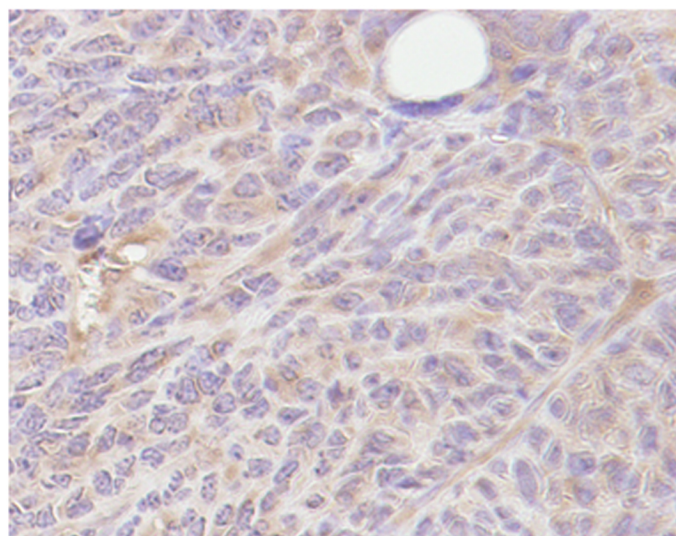

Control (IV)

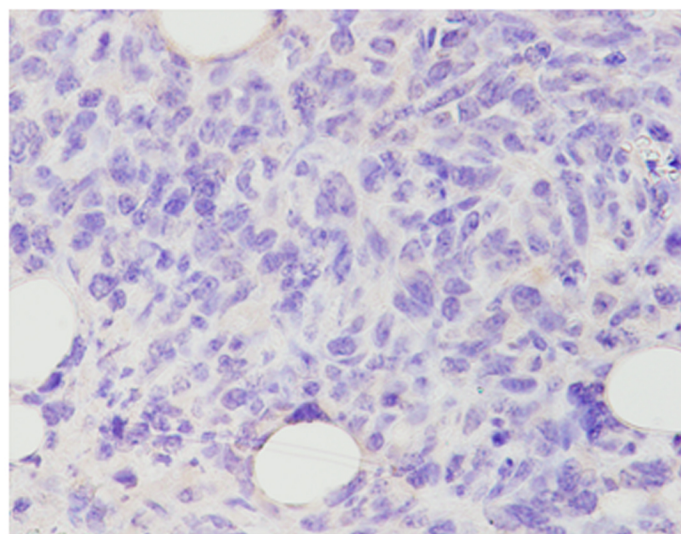

Supplement: Supplementary file 2 [file FBA2-2-5-s002.pdf]

**Expression of CD40L**

CD40L (IV)

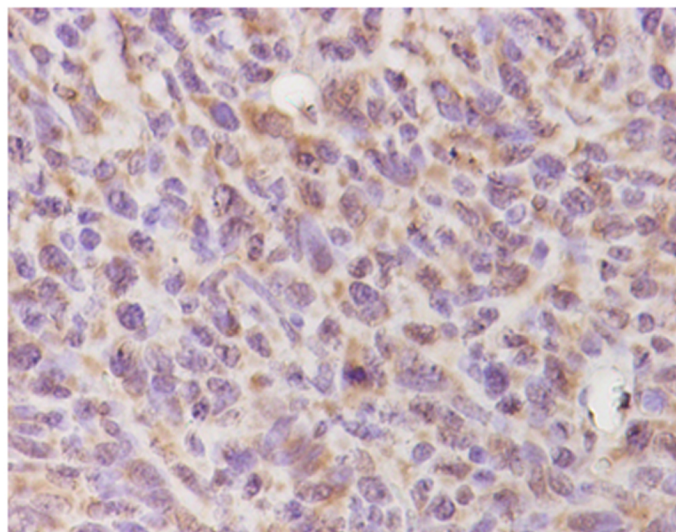

Control (IV)

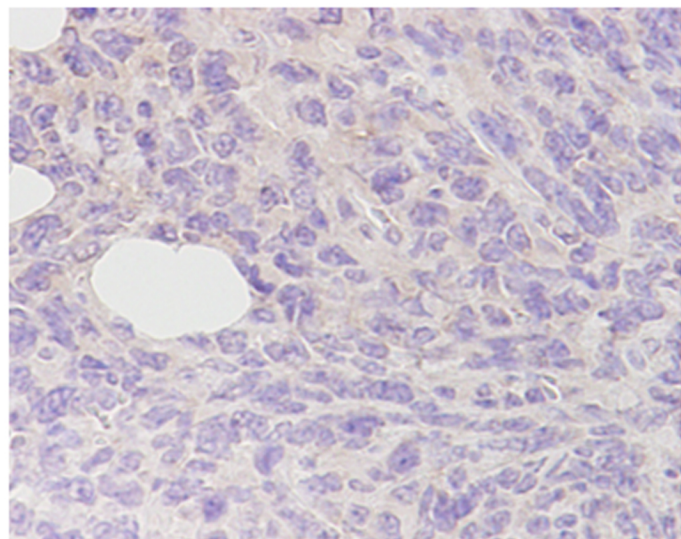

Supplement: Supplementary file 3 [file FBA2-2-5-s003.pdf]

**Mature DCs**

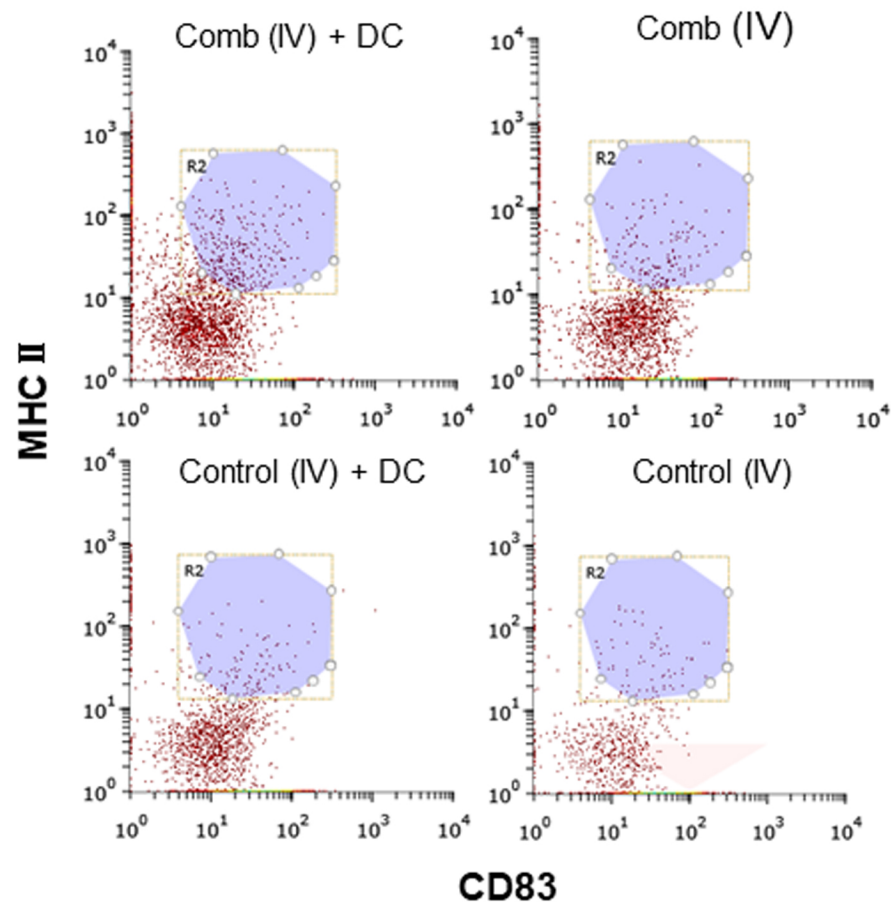

Supplement: Supplementary file 4 [file FBA2-2-5-s004.pdf]

Supplementary Figure S5

**NK cells**

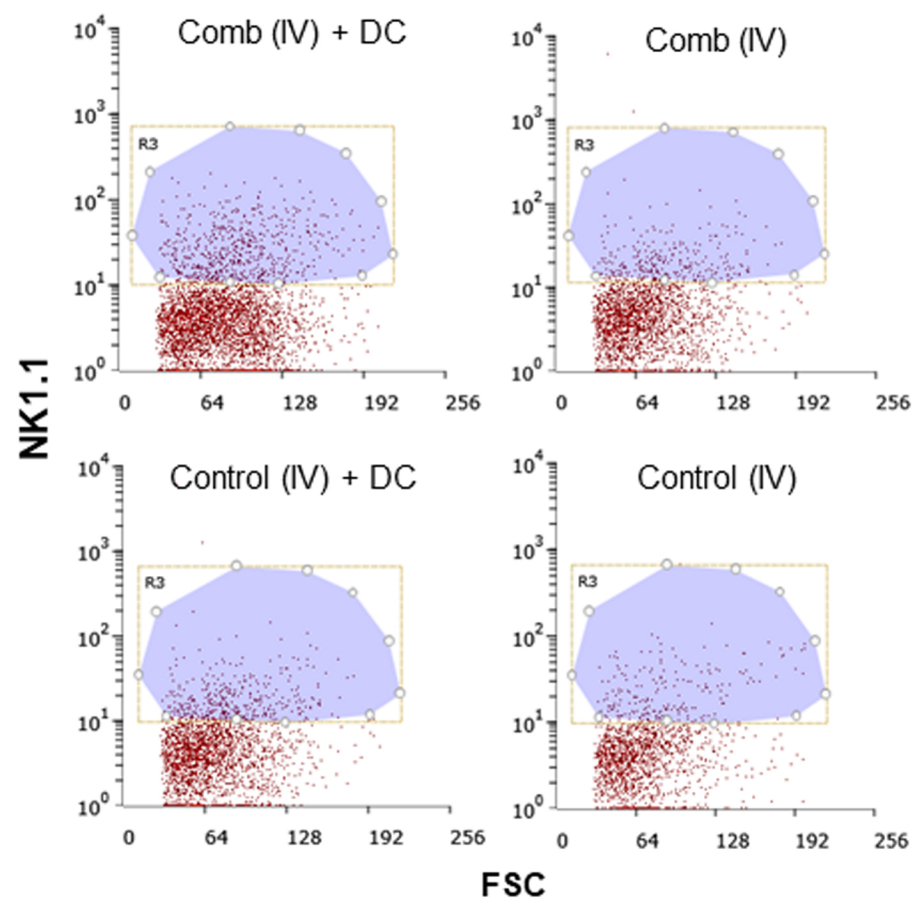

Supplement: Supplementary file 5 [file FBA2-2-5-s005.pdf]

Supplementary Figure S6

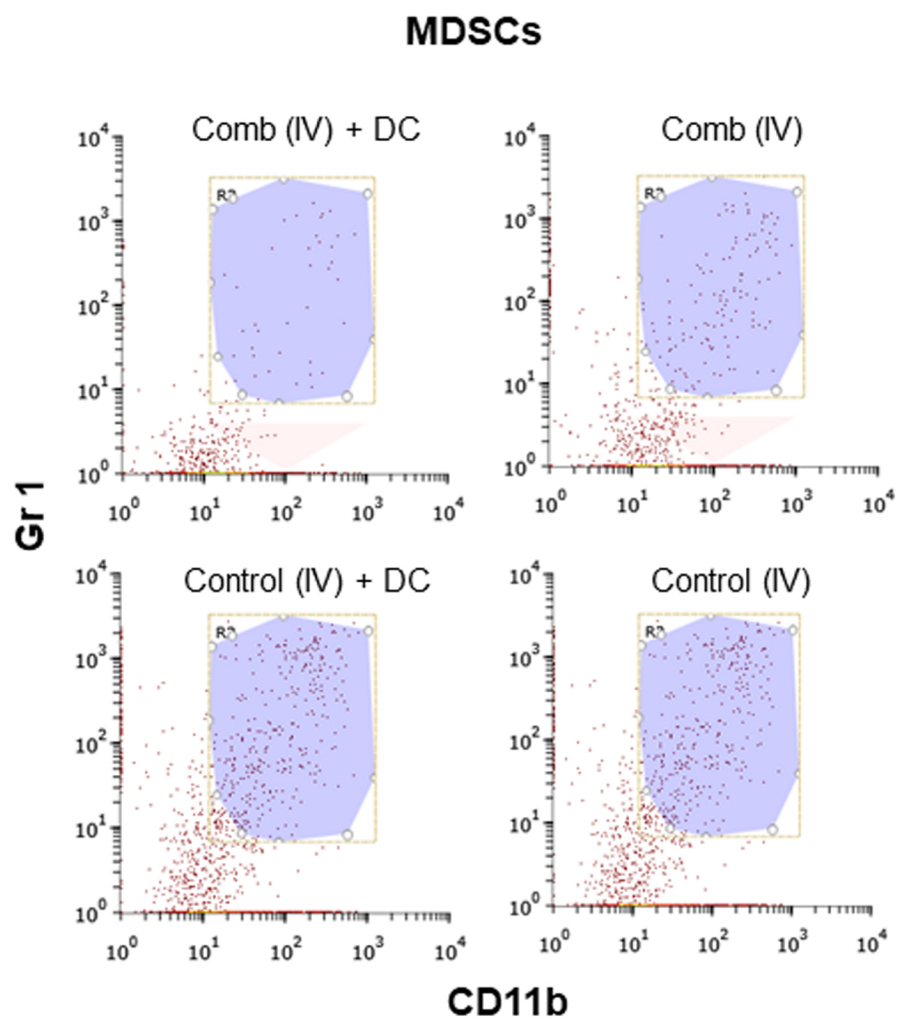

Supplement: Supplementary file 6 [file FBA2-2-5-s006.pdf]

Supplementary Figure S7

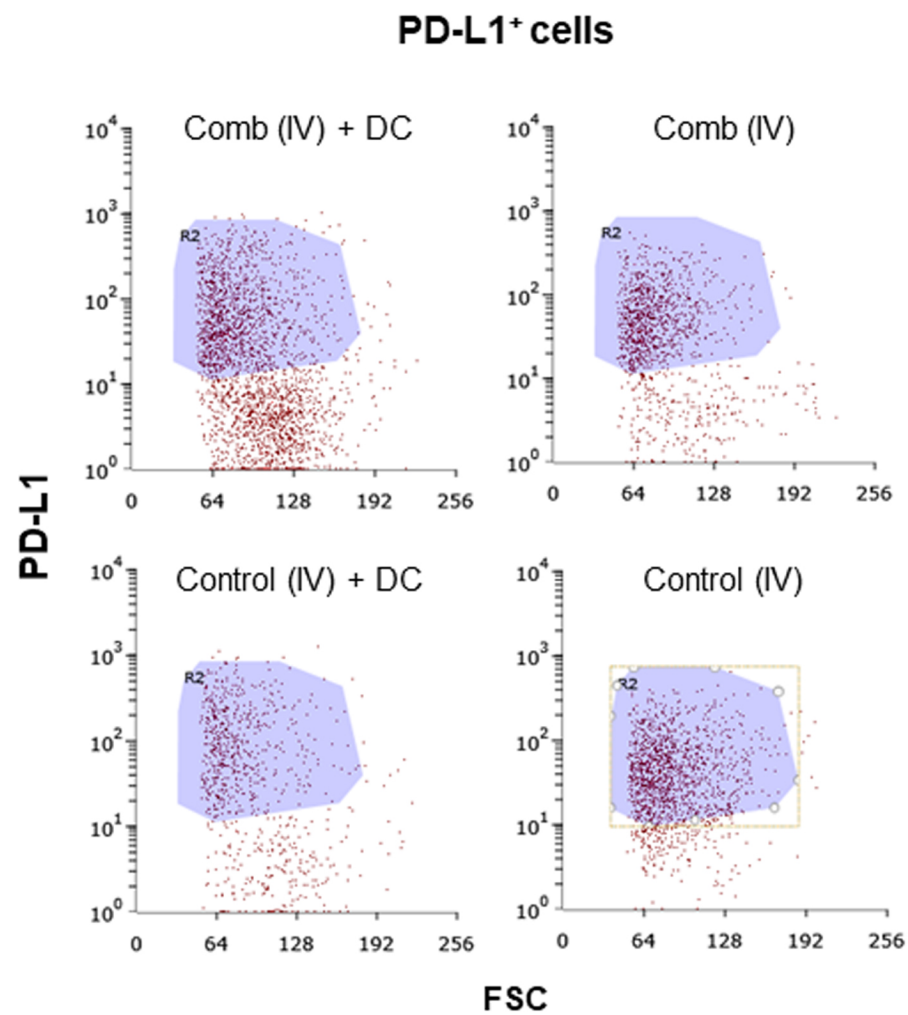

Supplement: Supplementary file 7 [file FBA2-2-5-s007.pdf]

Supplementary Figure S8

**Iba-1<sup>+</sup> cells**

Comb (IV) + DC

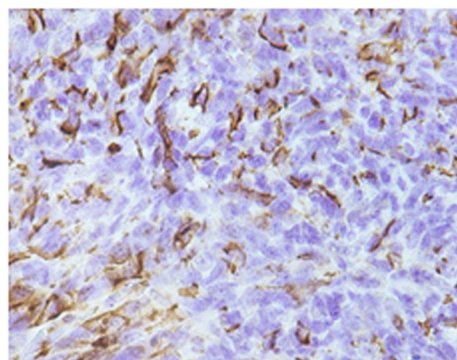

Comb (IV)

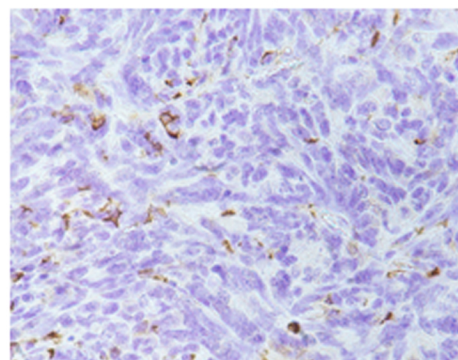

Control (IV) + DC

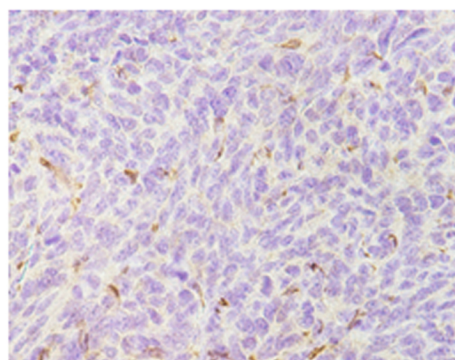

Control (IV)

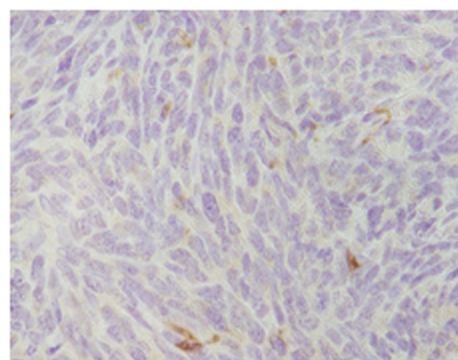

Supplement: Supplementary file 8 [file FBA2-2-5-s008.pdf]

Supplementary Figure S9

**Granzyme B<sup>+</sup> cells**

Comb (IV) + DC

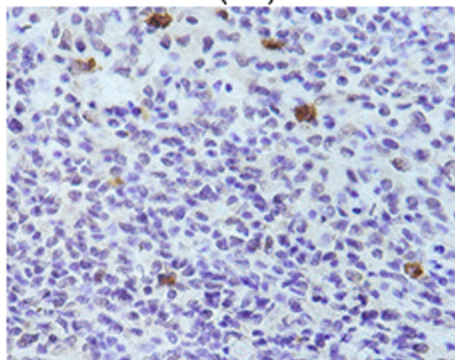

Comb (IV)

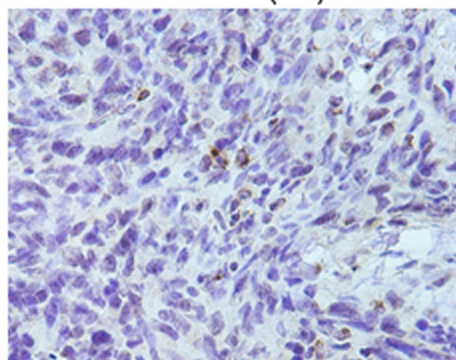

Control (IV) + DC

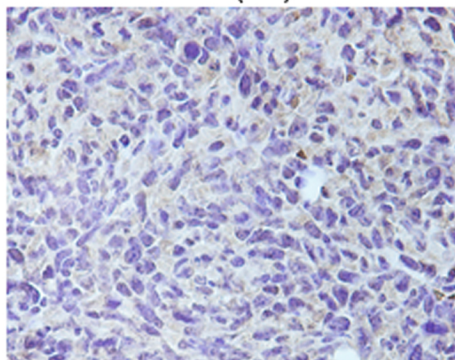

Control (IV)

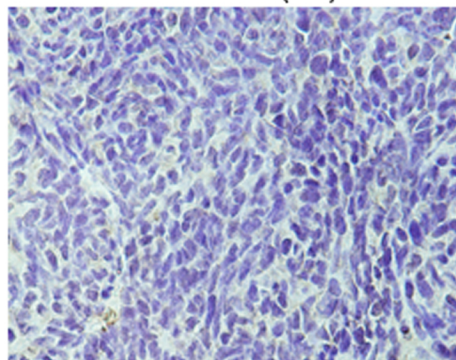

Supplement: Supplementary file 9 [file FBA2-2-5-s009.pdf]
