# Supplementary material for: Manipulation of the tumor microenvironment by cytokine gene transfection enhances dendritic cell‐based immunotherapy
Source: FASEB Bioadv. 2019 Nov 26;2(1):5–17. doi: 10.1096/fba.2019-00052 (PMC6996313; doi:10.1096/fba.2019-00052)
Supplement: Supplementary file 10 [file FBA2-2-5-s010.pdf]

LEGENDS OF SUPPLEMENTARY FIGURES

Figure S1 Expression of GFP in tumors in mice after intratumoral (IT) or intravenous (IV) injection of synthetic vehicle containing a GFP cDNA-plasmid. In GFP (IT) tissues, response was variable, ranging from strong (a) to very weak (b). All tissues were collected 2 days after treatment.

Figure S2 Expression of IFNγ in tumors in mice after intravenous (IV) injection of synthetic vehicle containing a IFNγ cDNA-plasmid or a Control cDNA-plasmid. Typical photos of tumors are shown. There were three mice per group and tissues were collected 2 days after treatment.

Figure S3 Expression of CD40L in tumors in mice after intravenous (IV) injection of synthetic vehicle containing a CD40L cDNA-plasmid or a Control cDNA-plasmid. Typical photos of tumors are shown. There were three mice per group and tissues were collected 2 days after treatment.

Figure S4 FCM profile of mature DCs in tumors in mice. Tissues were collected 7 days after the second treatment and analyzed to detect mature DCs (MHC II ^{high} CD83^{+} cells). Typical data are shown.

Figure S5 FCM profile of NK cells in tumors in mice. Tissues were collected 7

days after the second treatment and analyzed to detect NK cells (NK1.1^{+} cells). Typical data are shown.

Figure S6 FCM profile of MDSCs in tumors in mice. Tissues were collected 7 days after the second treatment and analyzed to detect MDCs (CD11b^{+}, Gr1^{+} cells). Typical data are shown.

Figure S7 FCM profile of PD-L1^{+} cells in tumors in mice. Tissues were collected 7 days after the second treatment and analyzed to detect PD-L1^{+} cells. Typical data are shown.

Figure S8 Immunohistochemistry profile of Iba 1^{+} macrophages in tumors in mice. Tissues were collected 7 days after the second treatment and analyzed to detect Iba 1^{+} cells. Typical photos are shown.

Figure S9 Immunohistochemistry profile of granzyme B^{+} killer cells in tumors in mice. Tissues were collected 7 days after the second treatment and analyzed to detect granzyme B^{+} killer cells. Typical photos are shown.
